# Laser, vacuum and gas reaction chamber for *operando* measurements at NSLS-II’s 28-ID-2

**DOI:** 10.1107/S160057752500829X

**Published:** 2025-10-24

**Authors:** Lauren Y. Moghimi, Patrik K. Johansson, Subhechchha Paul, Yifan Wang, Sara Irvine, Remington Graham, Deja Dominguez, Zane Taylor, Angel A. Martinez, John T. Markert, John Trunk, Hui Zhong, Jianming Bai, Sanjit Ghose, Leora Dresselhaus-Marais

**Affiliations:** a Department of Materials Science and Engineering Stanford University Stanford CA94305 USA; b PULSE Institute SLAC National Accelerator Laboratory Menlo Park CA95024 USA; c Department of Mechanical Engineering Stanford University Stanford CA94305 USA; d SIMES SLAC National Accelerator Laboratory Menlo Park CA95024 USA; e Department of Applied Physics Stanford University Stanford CA94305 USA; f Department of Physics University of Texas at Austin Austin TX78712 USA; g National Synchrotron Light Source II Brookhaven National Laboratory Upton NY11973 USA; RIKEN SPring-8 Center, Japan

**Keywords:** *operando* X-ray diffraction, transmission mode, single-shot experiments, lasers, vacuum conditions, *in situ* studies, powders, iron oxide reduction

## Abstract

We present a laser reaction chamber that we have developed for *in situ*/*operando*X-ray diffraction measurements at the NSLS-II 28-ID-2 X-ray powder diffraction beamline. This chamber allows for rapid and dynamic sample heating under specialized gas environments, spanning ambient conditions down to vacuum pressures. We demonstrate the capabilities of this setup through two applications: laser-driven heating in polycrystalline iron oxide and in single-crystal WTe_2_. Our measurements reveal the ability to resolve chemical reaction kinetics over minutes with 1 s time resolution. This setup advances opportunities for *in situ*/*operando* X-ray diffraction studies of both bulk and single-crystal materials.

## Abstract 2

A laser reaction chamber has been developed for *in situ*/*operando* X-ray diffraction measurements on the NSLS-II 28-ID-2 beamline, allowing for rapid sample heating under various gas and vacuum conditions. This capability has enabled the tracking of chemical reaction kinetics in polycrystalline iron oxide and a reversible phase transformation in WTe_2_ with 1 s time resolution, expanding the opportunities for dynamic studies in both bulk and single-crystal materials.

## 1. Introduction

Time-resolved measurements give insight into the mechanisms underlying materials synthesis and processing. Chemical kinetic parameters like reaction rate constants (*k*) are used to obtain activation energies (*E*_a_) in Arrhenius-type datasets, which provide insights into the reduction mechanisms and assist with process optimization. Reduction of solid-state iron oxides has been studied with *in situ* techniques including thermogravimetric analysis and temperature-programmed X-ray diffraction (XRD) to measure phase transition temperatures (Bulavchenko *et al.*, 2019 ▸; Qu *et al.*, 2014 ▸; Luu *et al.*, 2025 ▸). These reports, and similar studies, have been helpful to the H_2_-based reduction field by experimentally validating transition temperatures and rate-limiting steps in varied sample systems. However, such techniques cannot resolve phase distributions or reaction dynamics for changes on the order of seconds, or under non-equilibrium or *operando* conditions.

*In situ* reflection-mode XRD is a widely accessible technique to measure structural changes under sample heating, but its capability is often limited to sample surfaces due to small X-ray penetration depths relative to the sample width. In comparison to reflection-mode XRD, transmission-mode XRD has the benefit of enabling either bulk or surface analysis, depending on the sample geometry and scan configuration. Inherently, the interaction volume is larger in transmission-mode XRD, allowing users to capture microstructural results that are representative of a full sample rather than just the sample surface.

The high-energy X-ray beam (∼70 keV) provided by the 28-ID-2 beamline at the National Synchrotron Light Source II (NSLS-II) enables single-shot transmission-mode XRD measurements with a 1 s time interval. This includes *in situ* powder XRD measurements that can be used to study phase transitions on otherwise X-ray-opaque samples (Shi *et al.*, 2013 ▸). The adjustable beam size can be expanded to match the scale of heterogeneity in 1 mm-sized samples, or reduced to 100 µm to capture the local structural evolution on a slice of the sample. To date, the beamline has supported a variety of *in situ* and *operando* XRD experiments that benefit from instrumentation such as heating sources (lamp furnace, heating stage, coil heater, hot air blower) and specialized sample environments (Elbakhshwan *et al.*, 2016 ▸; Haas *et al.*, 2021 ▸; Avila *et al.*, 2021 ▸; Plonka *et al.*, 2019 ▸). The wide variety of instruments available on this beamline have impacted fields spanning battery materials, flash sintering and catalysis. For some applications in catalysis and chemistry, however, an additional reaction chamber capable of wavelength-tunable photochemistry with variable gas environments is required. Here we present an adaptable laser reaction chamber for measurements at the 28-ID-2 beamline, under rapid/dynamic sample heating and specialized gas environments, from ambient down to vacuum pressures. To demonstrate this setup, we present its use in two areas: (i) chemical reduction in polycrystalline iron oxides and (ii) phase transitions in single-crystal WTe_2_.

We show how this setup can resolve minutes-long chemical reactions, measured with ∼1 s time resolution. This setup can be useful for various *in situ*/*operando* applications investigating photo-induced transitions and laser-driven heating, such as photochemical catalysis, laser spot welding and pyrometallurgical minerals processing.

## 2. Design and implementation

**Figure 1:**
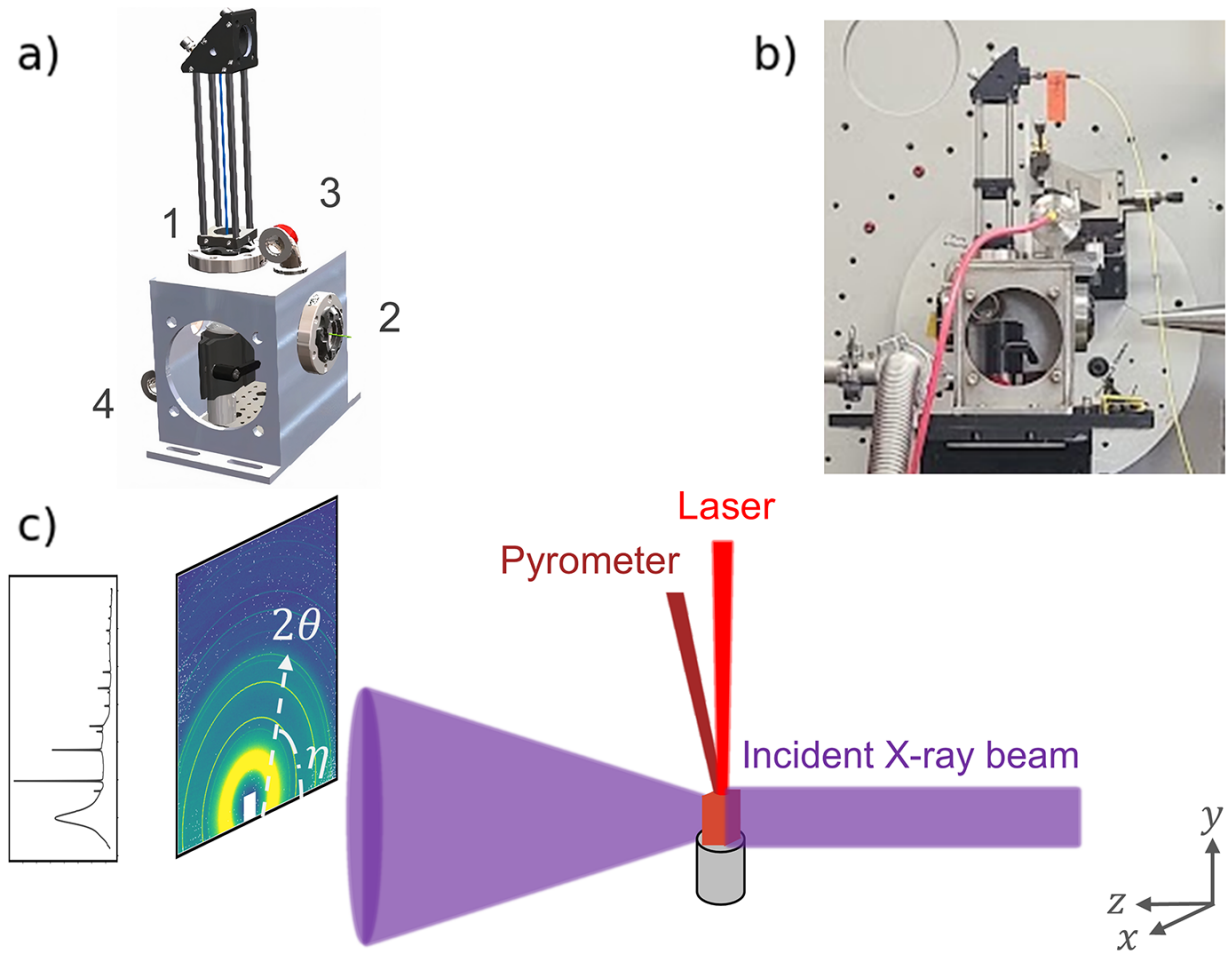
(*a*) The model and (*b*) a photograph of the reaction chamber for experiments at 28-ID-2. Labels 1, 2, 3 and 4 refer to the laser viewport, X-ray entry viewport, gas inlet, and gas outlet or vacuum port, respectively. The internal chamber is approximately 5.25 × 5.25 × 4 inches along the *x*, *y* and *z* directions. (*c*) Schematic of the measurement configuration for an iron oxide pellet sample.

We designed our reaction chamber to interface with beamline 28-ID-2, enabling XRD measurements up to 2θ = 15° at 68 keV in transmission mode. Figs. 1 ▸(*a*) and 1 ▸(*b*) show the model and a photograph of the reaction chamber, respectively.

### 2.1. Beamline specifications

The 2θ detection range and *q* resolution are two parameters that should be considered when deciding whether near-field or far-field diffraction measurements are more appropriate for an experiment. We utilize the beamline’s far-field detector, a PerkinElmer 1621, to achieve high *q* resolution. As a result, the size and position of the chamber’s X-ray exit port along the sample-to-detector distance limit the 2θ detection range. In experiments with the chamber at 68 keV, all phases of interest were resolvable in our mixed-phase samples and we accessed 2θ_max_ = 15°, which captured up to *q*_max_ = 10.5 Å^−1^. In terms of material parameters, values down to 0.095 Å that satisfy the diffraction condition can be reached by this *q* range. As this sufficiently captures the full range of *d* spacings for phase identification in most materials, Rietveld refinement may be used for quantitative phase analysis. This work uses the current beamline detectors that have the ability to record data at one shot per second, although planned detector upgrades (high-speed pixel counting) will soon support millisecond detection.

### 2.2. Chamber geometry and environment

To ensure that our chamber can operate under a variety of pressures, gas environments and high temperatures, we place all motors, sensors and optics outside of the chamber. The sample position relative to the X-ray beam is controlled by two mechanisms: (i) a motorized *xyz* stage outside the chamber and (ii) a manual sample mount inside the chamber. Our selected design eliminates the need for vacuum-compatible motors.

Chamber attachments are placed at ports facing the beamline-accessible side of the chamber stage (*i.e.* in +*x*) in case any modifications are needed while the chamber is mounted. KF16 flange elbows are welded onto the chamber for the gas inlet and outlet, enabling an adaptable setup such as switching between gas and vacuum inputs, or routing exhaust from the chamber. An optional digital vacuum gauge can measure the pressure within the chamber, and may be placed at or removed from the exit elbow.

Our chamber reached vacuum pressures as low as 0.04 torr. To ensure appropriate isolation of the effect of specific gas atmospheres on the iron oxide reduction behavior, we evacuated the chamber near this minimum pressure before introducing either 3% H_2_ in Ar or 100% Ar at a flow rate of 5 standard litres per minute.

### 2.3. Sample mounting

Sample size is selected based on the X-ray attenuation *versus* the integration volume of interest. For example, to study collective phenomena in a bulk sample, a large sample and wide beam size may be preferable to integrate heterogeneous processes uniformly. For sufficient transmission through all phases that emerged during the reduction from Fe_2_O_3_ (hematite), we limited the width of our powder samples to 2 mm or less. Our calculations showed that full-density pure Fe pellets at this width would have adequate transmissivity (23%) at 68 keV (Hubbell & Seltzer, 2004 ▸). To capture bulk reaction dynamics, we produced smaller 1 mm cubic samples to fit entirely within an expanded beam of 1.2 mm × 1.5 mm. Surfaces could also be studied using the same experimental configuration by restricting the overlap between the sample and X-ray beam to the surface.

We tested three forms of solid samples with this setup: a loose powder, a pelletized powder and a sheet of single crystal. Loose powder samples were mounted in rectangular cuvettes to contain the samples in rigid form. Fused quartz cuvettes with internal width of 2 mm and wall thickness of 0.75 mm were chosen to minimize parasitic contributions to the XRD signal. The cuvettes were positioned upright, such that the sample was directly exposed to the laser beam from above and the gas could pass over the sample freely [Fig. 1 ▸(*c*)].

We also prepared pellets of the hematite powder to reduce the risk of sample displacement away from the laser-illuminated spot and X-ray collection area, which occurred with powders, likely due to violent O_2_ off-gassing. We pre-mounted the samples onto dowel pins and fixed the position of the dowel holder to maintain consistent sample alignment across experiments. The dowel holder was a translating optical post assembly, which accommodated 0.635 mm of height adjustment per revolution.

Highly disordered single-crystal WTe_2_ samples were oriented with and without a tilt to observe microstructural transitions for diffraction planes of interest [*i.e.* along **c**, Fig. 5(*a*)]. Because of the high X-ray energy, not seen in previous studies, it was possible to transmit along the width of the sample (**b**) as well. We adapted a 3D-printed tilt mount to a vertically adjustable platform inside the reaction chamber and utilized two mounting geometries, where**b** was either 0° or 75° with respect to the X-ray beam axis (*z*).

For all samples, both the dowel holder and platform were manually adjusted in the vertical direction (*y*) and positioned in the *xz* plane. We fixed the mount position once we determined the desired locations during alignment. Fine control of the sample relative to the X-ray beam was achieved using a motorized *xyz* stage outside the chamber.

### 2.4. Alignment

After alignment of the upstream X-ray optics preceding the chamber, three steps were done before the setup was ready for well calibrated measurements:

(i) Position the chamber to the X-ray beam,

(ii) Align the laser beam to the X-ray beam,

(iii) Focus the pyrometer to the laser and X-ray beams.

Engineering controls and fixed sample mounting positions allowed us to maintain alignment between the three beams across experiments. We mounted the pyrometer and laser input to the *xyz* stage or directly to the chamber so that the pyrometer always measured the illumination spot on the sample surface.

**Figure 2:**
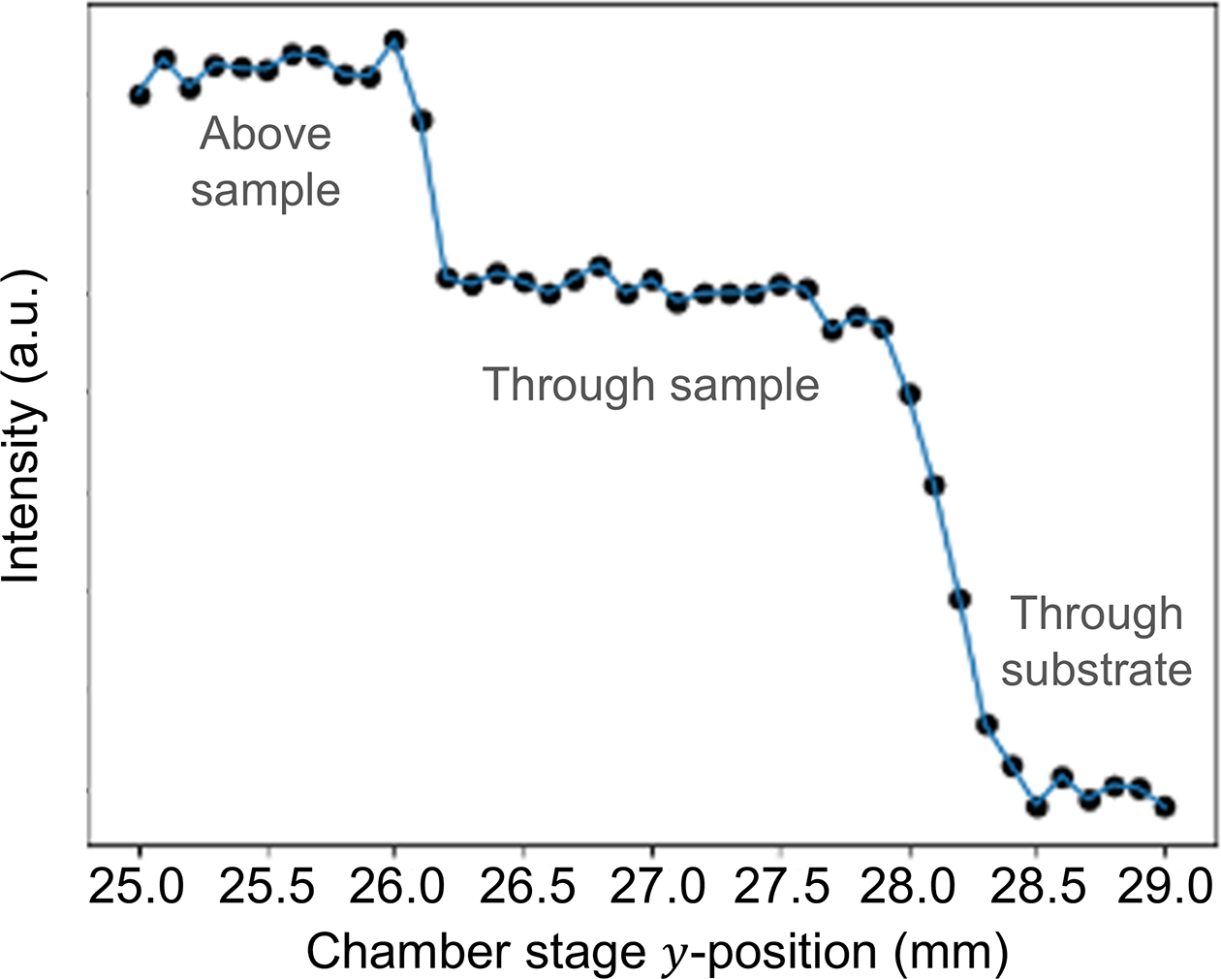
Transmitted X-ray intensity as a function of stage *y* position (mm) for an iron oxide pellet. From left to right, the plateaux correspond to the X-ray beam above the sample, through the sample and through the substrate.

In general, the chamber should be aligned with the X-ray beam in the *xy* plane so that the measurable 2θ range is maximized. Discontinuities along the detector’s azimuthal angle η can reveal crystalline texture, which can be especially important to capture in non-powder samples. As seen in Fig. 1 ▸(*c*), the 2D detector was offset above the X-ray axis so that the captured azimuthal range was just over Δη = 180°. To achieve up to 2θ_max_ = 15° with the azimuthal range as shown, we positioned the chamber by centering the X-ray viewports to the X-ray guide beam. We then adjusted the sample mount and the sample to the X-ray guide beam in *y*. We refined the chamber’s alignment to the X-ray beam by measuring the transmitted X-ray intensity while scanning the vertical motor position, as seen in Fig. 2 ▸. Before setting the chosen height for the *xyz* stage, we checked that we could measure sufficient sample diffraction at the selected position.

To measure the sample region under laser illumination with XRD, we next aligned the laser beam in the *xz* plane to the X-ray guide beam. Ideal alignment of the laser beam has the laser at an angle of 90° with respect to the X-ray beam, illuminating the sample on the top. For safety, we used the minimum power during laser alignment and higher power only via remote control after the hutch was unoccupied by personnel. We used a near-infrared (NIR) viewing card mounted on a viewport cover to help align the NIR laser beam. We aligned and fixed the *xz* position of the sample mount to the intersection of the laser and X-ray beams.

Finally, we aligned the pyrometer to the sample surface to measure the temperature at the laser-illuminated spot. The pyrometer has two 635 nm guide beams to assist with alignment; the beams cross at the device’s focal position. The collection area is given by the spot size, which is nominally 0.3 mm at the focal length (150 mm). The pyrometer was positioned at a grazing angle, which increased the collection area on the sample surface. Beyond alignment, we discuss the pyrometer specifically in Section 2.6.

### 2.5. Laser integration

**Figure 3:**
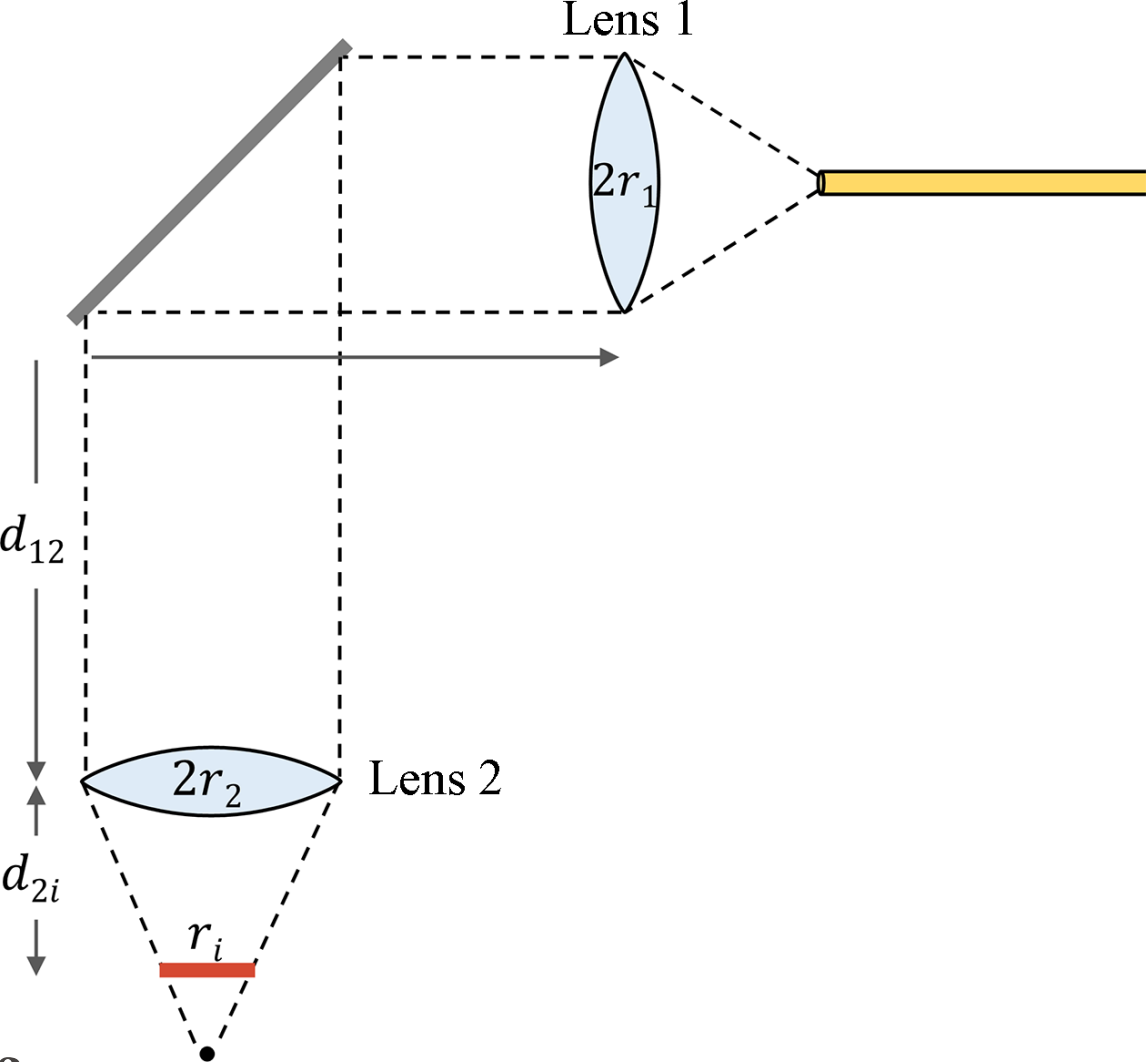
Schematic of the optics along the laser beam path, used to calculate the incident spot size *r*_*i*_. The beam radius just after lens 1 is *r*_1_ = 2 mm. The focal length of lens 2 is *f* = 100 mm. The distances *d*_12_ and *d*_2*i*_ are 170 mm and 75 mm, respectively.

The laser in this setup can be used to drive photochemical or thermal phase transitions, although the samples studied in this work were limited to temperature-driven transitions. Samples were heated by exposure to a continuous-wave 10 W fiber-coupled laser. The laser beam was guided along a cage system that mounted onto the chamber so that alignment was independent of chamber movement. We tuned the incident spot size using a fiber collimator and focusing lens that were compatible with the laser wavelength and using the setup shown in Fig. 3 ▸. The mirror was at a 45° tilt from the optical axis and had high reflectivity (97%) at the laser wavelength. To maximize the laser power transmitted through the laser viewport, we chose an anti-reflective window that spanned the 976 nm (NIR) and 445 nm (blue) wavelengths used thus far.

The spot size at the sample surface was determined by the following equations, where ϕ/2 = 0.018° is the divergence half-angle from lens 1. The beam diameter at lens 2 is given by
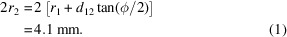
Similarly, the incident beam diameter is given by
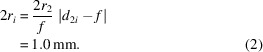
The electrical input for the laser was remotely controlled and digitally logged using the desktop control system provided by the power supply manufacturer (BK Precision 9103). Thus, our experiments were able to track incident optical power as a function of time. We calibrated the electrical power to the optical power illuminating the sample using a power meter.

### 2.6. Measurements of laser heating

We tracked the sample’s surface temperature remotely and *in situ* using a pyrometer (Micro-Epsilon CTLM-3H3CF2-C3), which offered several advantages over thermocouple feedthrough measurements. Optical pyrometer measurements were preferred in this work so that physical contact with the sample was not necessary. Non-invasive temperature measurements offer opportunities to study processing environments that may degrade thermocouples, such as chemically reactive, high-temperature or low-pressure conditions. We focused the pyrometer to the sample surface following the alignment scheme discussed in Section 2.4. We mounted a 1 µm longpass filter to prevent interference from our NIR laser with the temperature measurements. We used a CaF_2_ window for the pyrometer viewport to ensure high IR transmittance (>90%) up to a wavelength of 7 µm.

The device’s temperature readouts are between 250 and 1800°C. To calibrate the pyrometer after measurements, we calculated the temperature of heated pellets using the shift in diffraction peak position, as quantified by
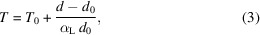
where α_L_ is the linear expansion coefficient, *T* is the sample temperature, *d* is the interplanar spacing at this temperature, and *T*_0_ and *d*_0_ are the respective values at room temperature. We additionally prepared pellets of 33 wt% CeO_2_ powder in hematite as a calibration standard for temperature, as CeO_2_ (ceria) is a NIST standard for quantitative XRD analysis. We chose this ceria-to-hematite ratio to preserve the pellet’s optical and thermal properties, while ensuring sufficiently high intensity of ceria’s main XRD peak within the hematite matrix. We note that the pyrometer was used to track the relative surface temperature during experiments for on-site analysis. XRD peak shifts were used to determine the true sample temperature averaged over the X-ray interaction volume.

## 3. Example applications

In this section, we discuss experiments on pellet and single-crystal samples that we studied using our laser reaction chamber. We plot everything in *q* such that our peaks are well resolved and photon energy-invariant, for comparison with other studies and materials.

### 3.1. Gas–solid reaction of pelletized hematite

We begin by presenting a study of hematite pellet reduction in various gas environments during laser irradiation. Hematite is among the iron oxides that are established chemical and photo-catalysts (Leland & Bard, 1987 ▸; Khedr *et al.*, 2009 ▸; Pang *et al.*, 2016 ▸; Parkinson, 2016 ▸; Shaikhutdinov *et al.*, 1999 ▸), with applications spanning water splitting (Badia-Bou *et al.*, 2013 ▸), water oxidation (Kanazawa & Maeda, 2017 ▸) and nitrate splitting (Jung *et al.*, 2014 ▸). We used our setup to identify hematite’s reduction pathway under H_2_, Ar and vacuum. As a demonstration, we focus on one experiment in H_2_.

**Figure 4:**
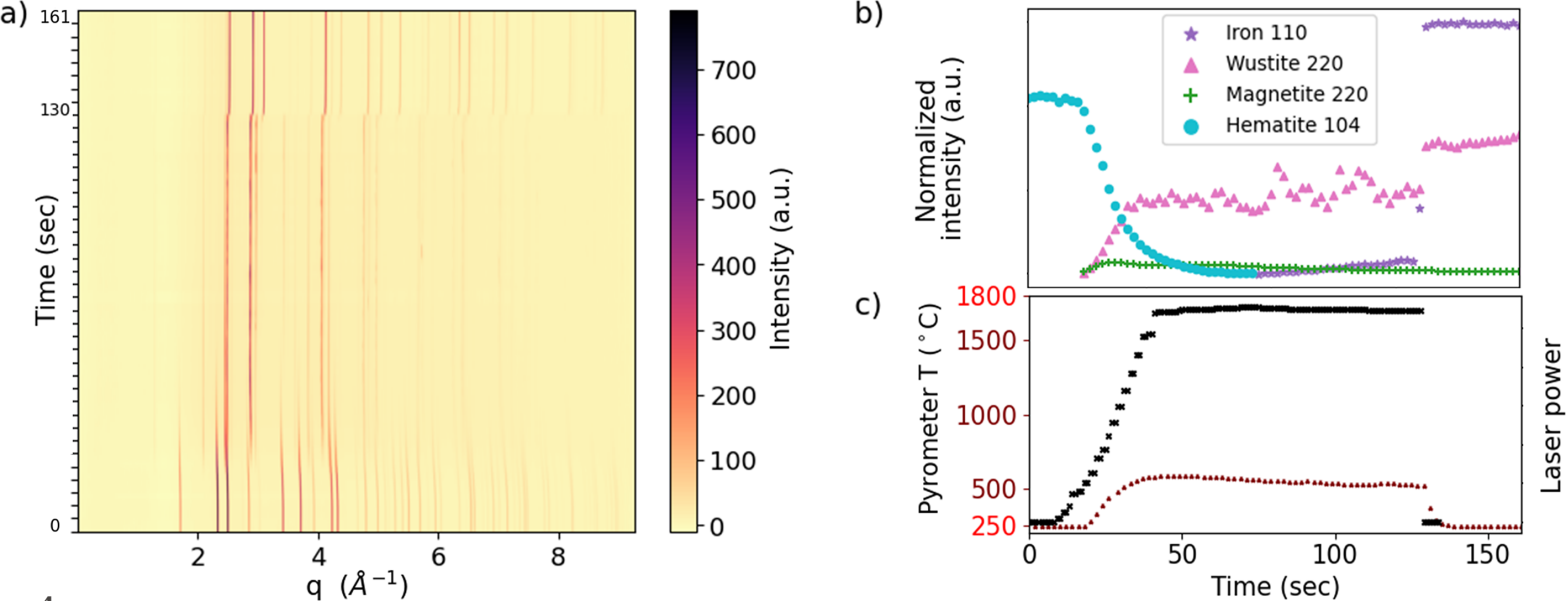
Data from *operando* hematite experiments in 3% H_2_ and laser heating with λ_laser_ = 976 nm, reaching a maximum power *P*_max_ = 10 W at the sample surface. (*a*) Waterfall plot of the background-subtracted 1D X-ray powder diffraction *versus* time. (*b*) The integrated intensity of one characteristic peak per phase *versus* time. (*c*) Plot of the laser input and the raw pyrometer output. The pyrometer’s operating range, 250–1800°C, is highlighted.

Figs. 4 ▸(*a*) and 4 ▸(*b*) show the raw and processed data, respectively, for hematite pellets heated in the 3% H_2_ environ­ment. We quantified each phase by integrating one well isolated peak and normalizing by its structure factor and multiplicity, as done in previous work (Zheng *et al.*, 2023 ▸). As shown by the laser power *versus* temperature plots in Fig. 4 ▸(*c*), the reactions in the hematite sample exhibited three dominant stages at the times when the laser was ramped, held at maximum power and shut off [Fig. 4 ▸(*b*)]. We note that the Debye–Waller effect is at least partially responsible for the increase in intensity at shutoff. Thus without temperature normalization, phase quantities can be compared only across isothermal periods. The sample reached at least 582°C at the surface during heating with maximum laser power. The heating laser also enabled fast heating rates, bringing samples to a steady-state temperature within 2 s at constant laser power.

This experiment has allowed us to observe the dynamics of iron oxide reduction in samples heated to high temperatures. Thermogravimetric analysis and temperature-programmed XRD studies have shown clear step-wise reduction following Fe_2_O_3_ → Fe_3_O_4_ → FeO → Fe (Jozwiak *et al.*, 2007 ▸). These studies hence produce reaction rates for each step-wise transition, or produce the conversion degree for the overall progression towards iron (Spreitzer & Schenk, 2019 ▸; Xing *et al.*, 2020 ▸). Our results show an even more complicated picture for reduction under non-equilibrium conditions (*i.e.* faster heating rates), wherein multiple phases are consumed and formed at each stage.

Importantly, to obtain quantitative kinetic parameters from measurements, the full sample system must be measured as a function of time. Hence we produced sample pellets to fit entirely within the integrated volume of the expanded 68 keV X-ray beam. We also aligned the lower boundary of the X-ray beam to the base of the pellets because we observed significant sample shrinkage during the experiments. While the quantitative kinetic parameters for these measurements are beyond the scope of this work, our setup lays the experimental foundation for fundamental sample studies under dynamic processing conditions, which can find use in other applications such as catalysis.

### 3.2. Reversible temperature-induced phase transition of single crystals

This section presents a sample experiment to demonstrate the chamber’s utility in studying a phase transition in single-crystal samples under vacuum. WTe_2_ is a quasi-2D material that has a phase transition from the room-temperature orthorhombic phase (*T*_d_) to the monoclinic (1*T*′) phase, which can be driven by temperature (Tao *et al.*, 2020 ▸), laser excitation (Sie *et al.*, 2019 ▸) or high pressure (Zhou *et al.*, 2016 ▸). The transition has been observed at ∼565 K, though different transition temperatures have been reported in single-crystal samples compared with polycrystalline powder samples (Tao *et al.*, 2020 ▸). Existing measurements have employed *in situ* neutron scattering and *in situ* powder XRD to study this phase transition. However, to the best of our knowledge, no previous study has utilized such a broad *q* range as presented in this work.

**Figure 5:**
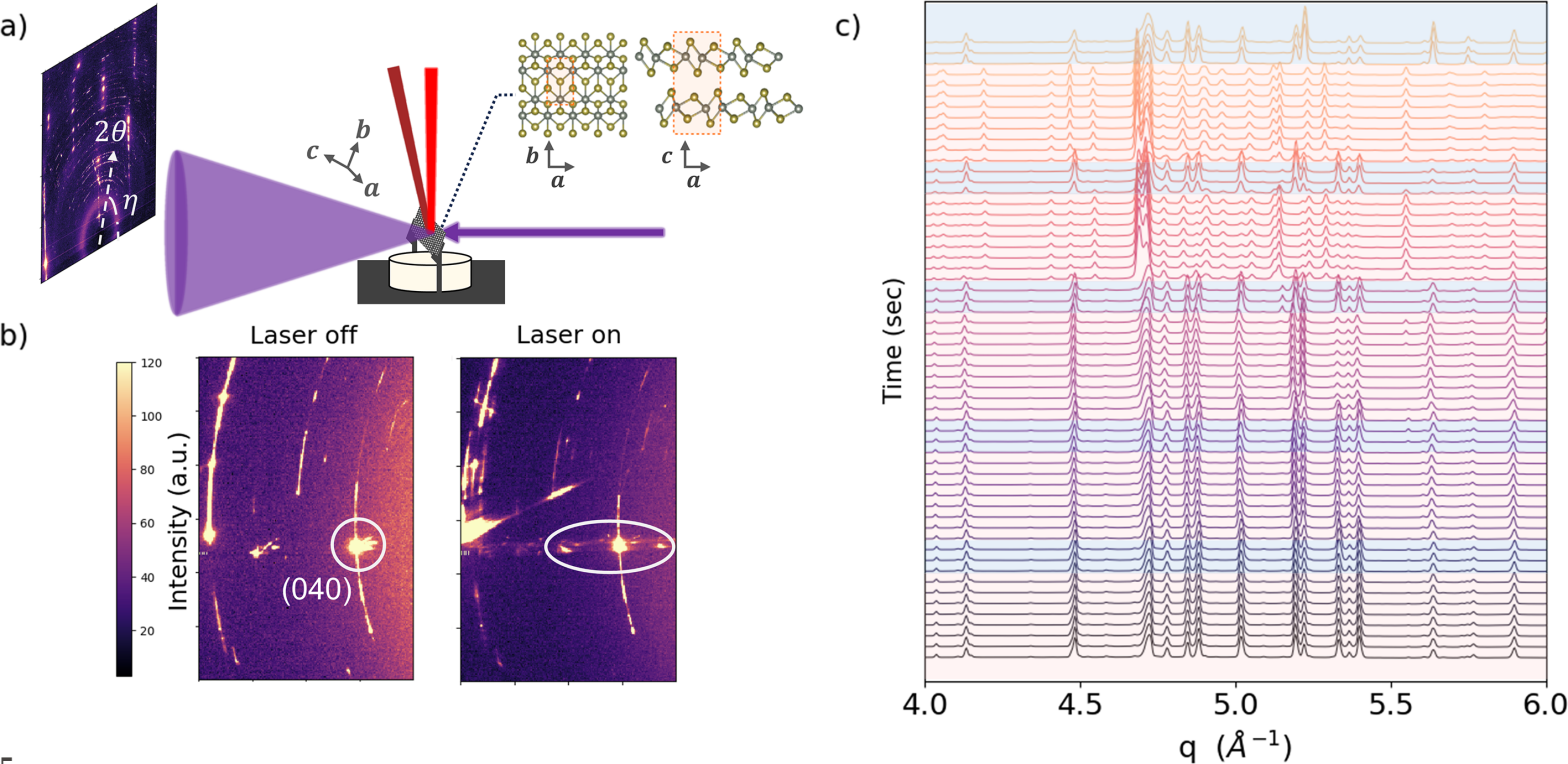
(*a*) Schematic of the WTe_2_ structure showing the crystal’s planar view, the side view where planes stack along **c** and the mounting geometry with respect to the X-ray beam for a tilted sample. We present the single-crystal diffraction data for a sample where **b** is parallel to the X-ray beam, to showcase the transition during laser on/off in (*b*) the 2D diffraction data and (*c*) the 1D diffraction data. Here the diffraction patterns are plotted in a waterfall format to reveal subtle changes in the peak profile as the laser is turned on/off and the temperature increases towards the transition point. Scans with the laser on are highlighted in red, while those with the laser off are highlighted in blue. The time step per scan is 5.2 s.

In this work, we used a 976 nm laser to thermally drive the *T*_d_-to-1*T*′ transition. Disordered single-crystal samples were prepared by chemical vapor transport (CVT) with subsequent exfoliation, and were ∼4 mm × 1.5 mm × 1 mm in the **a**, **b** and **c** directions, respectively. Enabled by this setup and the 68 keV X-ray energy, we oriented the sample to transmit partially or entirely along **b**, which allowed for the peaks most sensitive to the phase transition to meet the diffraction condition [Fig. 5 ▸(*a*)]. XRD measurements along this axis are uncommon due to the material’s high X-ray attenuation and to challenges with cleaving bulk samples to reduced thicknesses.

We note that the sample of interest in this study is a disordered single crystal, hence we observe crystal texture in the starting sample. As the laser power rises, the *T*_d_ sample increasingly forms 1*T*′, as shown by the crystal diffraction patterns (Fig. 5 ▸). Fig. 5 ▸(*b*) reveals laser-induced texturing that emerges from the (040) peak, and peak profile broadening and peak formation can be seen in Fig. 5 ▸(*c*). While we plot the diffraction traces, we avoid labeling peaks for each phase because the lattice parameters are not well defined for these phases. As this transition has not been driven by laser heating in previous research, there is uncertainty around the transition temperature. However, the combination of the heating laser, vacuum environment, high X-ray energy and broad *q* range in this experiment provides the framework for high-temperature heating under vacuum for single-crystal samples.

## 4. Discussion

The reaction chamber developed in this work enabled 1 s resolved measurements of phase changes induced by reactive gas and laser heating. Using a laser as the heating source and with remote sensors, we achieved rapid heating rates without compromising the measurements due to high temperatures. Surface temperatures beyond 1250°C were detected with the pyrometer in various environments: H_2_, Ar and vacuum. Future users may change the NIR laser for other wavelengths or powers by adjusting the optics accordingly. The current layout ensures that the laser and pyrometer are always aligned with the sample surface, even when the chamber stage is moved to change the XRD measurement location. Additionally, users interested in measuring pristine single-crystal samples may want to consider including a rotation stage inside the reaction chamber.

As discussed above, the capabilities of this chamber offer two modalities. Quantitative phase analysis was demonstrated with 1 mm iron oxide pellets that fit entirely within the beam for the duration of the experiment. This was enabled by integrated intensity phase analysis and by the fact that conservation of iron could be assumed. The other modality can study heterogeneity using a beam that is smaller than the sample size and focused on a selected sample region. This modality could also support depth-resolved temperature measurements and the impact of the heat distribution on the reaction profile. Importantly, samples with fixed thermal volume during laser heating would be well suited for depth-resolved measurements.

Future upgrades of this setup are ongoing to enhance further the types of material dynamics that can be studied with this chamber. The most imminent of these are detector upgrades that may enable sub-second measurements. To enable the timing precision for those types of sub-second measurements, we are working to synchronize the laser with the existing X-ray/pyrometer measurements accordingly. Additional efforts are working to incorporate a vacuum-compatible *xyz* motorized sample stage and partial pressure probes for reactive gases (*e.g.* O_2_, H_2_*etc.*).

## 5. Conclusion

In this work, we have presented a multi-purpose and adaptable reaction chamber for transmission-mode XRD. The chamber capabilities include controlled gas flow up to 5 standard litres per minute, vacuum pressures down to 0.04 torr, remote temperature sensing and up to 10 W of laser power with 1 s XRD acquisition times. To demonstrate the setup, we have presented NIR laser-driven heating measurements in two sample types: (i) powder iron oxide reduction with H_2_ and (ii) single-crystal WTe_2_ under vacuum. Experiments reached at least 1250°C and achieved the associated phase transition and chemistry. The reaction chamber setup described here expands opportunities to study high-*Z* and thick samples *operando* and *in situ* in the X-ray powder diffraction (NSLS-II 28-ID-2) hutch.

## Supplementary Material

Supporting information: Viewport windows. DOI: 10.1107/S160057752500829X/yi5177sup1.pdf

## Data Availability

The raw data presented in this work are available at https://drive.google.com/drive/folders/1UPx2exhB2RwePGqRC3scFO-sBXupoMUF?usp=sharing and were analyzed by code available at https://github.com/leoradm/NSLS-II-Methods-2025.
