# Supplementary material for: Laser, vacuum and gas reaction chamber for operando measurements at NSLS-II’s 28-ID-2
Source: J Synchrotron Radiat. 2025 Oct 24;32(Pt 6):1561–8. doi: 10.1107/S160057752500829X (PMC12591078; doi:10.1107/S160057752500829X)
Supplement: Supplementary file 1 [file s-32-01561-sup1.pdf]

JOURNAL OF SYNCHROTRON RADIATION

**Volume 32 (2025)**

**Supporting information for article:**

# Laser, vacuum, and gas reaction chamber for *operando* measurements at NSLS-II's 28-ID-2

**Lauren Y. Moghimi, Patrik K. Johansson, Subhechchha Paul, Yifan Wang, Sara Irvine, Remington Graham, Deja Dominguez, Zane Taylor, Angel A. Martinez, John T. Markert, John Trunk, Hui Zhong, Jianming Bai, Sanjit Ghose and Leora Dresselhaus-Marais**

### S1. Viewport windows

Depending on the user's experimental needs, a user might want to replace the window to reduce the XRD background/signal attenuation, find a compatible window when the laser or pyrometer operation wavelength is changed, or reach lower vacuum pressures. The VC22FL and VC23FL viewport mounts allow the windows to be switched out on-site without replacing a full flange with a pre-mounted window. We used 0.5 mm-thick x 1 inch-diameter fused quartz for the X-ray entry window and 1 mm-thick x 1.5 inch-diameter for the exit window. We calculate 1 mm thick $SiO_2$ has 72% transmittance at 68 keV. One can replace the windows for a different material and thickness as needed, so long as the window will withstand the internal chamber pressure during experiments. One should consult the following equation, which governs the minimum window thickness required to withstand the pressure differential between atmosphere and vacuum,

$$t = 1.1\, P\, r^2\ \mathrm{SF} / \mathrm{MR},$$

where $P$ is the pressure difference in psi, $r$ is the unsupported radius of the window, SF is the safety factor – typically between 4 and 6 – and MR is the modulus of rupture in psi. One should also consider the X-ray attenuation through both X-ray windows, the sample and the sample holder (if applicable), depending on the sample composition and magnitude of microstructural changes that are to be measured. The maximum window thickness that can be supported by the 1 inch and 1.5 inch viewport mounts are 5 mm and 4 mm, respectively. Since our windows were thinner than the space available in the viewport mounts, we 3D-printed spacer rings to maintain a gas-isolated system inside the chamber.
